# Forests trapped in nitrogen limitation – an ecological market perspective on ectomycorrhizal symbiosis

**DOI:** 10.1111/nph.12840

**Published:** 2014-05-14

**Authors:** Oskar Franklin, Torgny Näsholm, Peter Högberg, Mona N Högberg

**Affiliations:** 1 IIASA- International Institute for Applied Systems AnalysisA-2361, Laxenburg, Austria; 2 Department of Forest Ecology and Management, Swedish University of Agricultural SciencesSE-901 83, Umeå, Sweden

**Keywords:** below-ground, elevated carbon dioxide, game theory, mutualism, optimization, pine, trade, tragedy of the commons

## Abstract

- Ectomycorrhizal symbiosis is omnipresent in boreal forests, where it is assumed to benefit plant growth. However, experiments show inconsistent benefits for plants and volatility of individual partnerships, which calls for a re-evaluation of the presumed role of this symbiosis.
- We reconcile these inconsistencies by developing a model that demonstrates how mycorrhizal networking and market mechanisms shape the strategies of individual plants and fungi to promote symbiotic stability at the ecosystem level.
- The model predicts that plants switch abruptly from a mixed strategy with both mycorrhizal and nonmycorrhizal roots to a purely mycorrhizal strategy as soil nitrogen availability declines, in agreement with the frequency distribution of ectomycorrhizal colonization intensity across a wide-ranging data set. In line with observations in field-scale isotope labeling experiments, the model explains why ectomycorrhizal symbiosis does not alleviate plant nitrogen limitation. Instead, market mechanisms may generate self-stabilization of the mycorrhizal strategy via nitrogen depletion feedback, even if plant growth is ultimately reduced.
- We suggest that this feedback mechanism maintains the strong nitrogen limitation ubiquitous in boreal forests. The mechanism may also have the capacity to eliminate or even reverse the expected positive effect of rising CO_2_ on tree growth in strongly nitrogen-limited boreal forests.

## Introduction

The combination of vast carbon (C) stores and a particularly strong projected temperature rise makes boreal forests a critical component of the future climate system (Foley *et al*., 1994). While low nitrogen (N) supply typically constrains C input by plant growth in this biome (Jarvis & Linder, 2000), large amounts of C are stored in the soils (Post *et al*., 1982). Although well-known factors such as net primary production, temperature, and precipitation all influence C storage in forest soils, there is an even stronger predictor of high soil C – the presence of ectomycorrhiza (Averill *et al*., 2014). This fundamental component of forest ecosystems is highly sensitive to ongoing global changes such as rising atmospheric CO_2_ (Fransson *et al*., 2005; Garcia *et al*., 2008) and N deposition (Cox *et al*., 2010; Högberg *et al*., 2010; Bahr *et al*., 2013). Not only ectomycorrhizal fungi (EMF) but also arbuscular mycorrhizal fungi (AMF) have the potential to significantly influence forest N dynamics (Hodge & Fitter, 2010). Consequently, the stability and behavior of mycorrhizal symbiosis in forests may play a significant role in the progression of global change. However, evaluation of mycorrhizal responses to global changes is hampered by our limited mechanistic understanding of the role of mycorrhizal symbiosis in ecosystems (Johnson *et al*., 2013). Here we aim to establish principles for the stability and function of ectomycorrhizal symbiosis in a forest ecosystem context, focusing on boreal forests.

Evolution of symbiosis between N-limited plants and C-limited mycorrhizal fungi should require mutualistic C–N exchange (Hoeksema & Schwartz, 2003); that is, both parties benefit by participating in the exchange. However, plants do not always benefit (Egger & Hibbett, 2004; Jones & Smith, 2004; Corrêa *et al*., 2012; Walder *et al*., 2012). Even in strongly N-limited boreal forest, a recent study suggests that EMF sustain rather than alleviate plant N limitation by reducing the fraction of fungal N uptake transferred to trees as soil N availability declines (Näsholm *et al*., 2013). Conversely, experimental N additions increased the proportion of N transferred to the trees and the N : C exchange ratio between fungi and trees, implying a greater symbiotic benefit for the trees at high than at low soil N availability (Näsholm *et al*., 2013). These findings appear inconsistent with the common view of ectomycorrhizal symbiosis and its predominance in nutrient-poor forests.

Recent theoretical progress on mycorrhizal symbiosis has elucidated the conditions for evolutionary stability of pair-wise partnerships in the presence of nonsymbiotic alternative strategies and potential cheaters, that is, individuals taking benefits without providing something in return to their partners (de Mazancourt & Schwartz, 2010; Grman *et al*., 2012). However, these models are not obviously applicable to the multiple-partner structure of ectomycorrhizal symbiosis and its dynamic nature (Kennedy, 2010; Pickles *et al*., 2010). More specifically, in addition to the inconsistent benefits for plants discussed above, lack of partner specificity and fidelity in ectomycorrhizal symbiosis (Kennedy, 2010) challenge mycorrhizal theories based on evolved species-specific pair-wise partnerships. An ecological market perspective (Noë & Hammerstein, 1994; Jones *et al*., 2012; Werner *et al*., 2014) opens a way forward by allowing mutualistic symbiosis to be maintained by market mechanisms, such as optimal selection of trading partners (a host distributes trading among its partners to minimize overall ‘cost’ per unit resource (Kummel & Salant, 2006)) and reciprocal rewards, that is, beneficial behavior toward a partner is reciprocated (Kiers *et al*., 2011; Fellbaum *et al*., 2012). However, observations of strongly nonreciprocal behavior (Walder *et al*., 2012) appear to call for another explanation. More generally, the ecologically critical questions remain: why does mycorrhizal symbiosis often persist even when it appears not to benefit plants, and what are the consequences of this behavior for ecosystem function? We hypothesize that the interactions among multiple partners of both plants and fungi (mycorrhizal networks) shape the strategies of individuals to promote symbiotic stability even if forest productivity is reduced. The hypothesis is implemented as a tractable market model of C–N exchange in boreal forest, which goes beyond earlier market models (e.g. Kummel & Salant, 2006) by accounting for multiple simultaneous partners of both plants and fungi and adaptation of individuals’ strategies. We evaluate the model against published empirical observations, and discuss its implications for the role of mycorrhizal symbiosis in the boreal forest and more generally.

## Description

**Figure 1:**
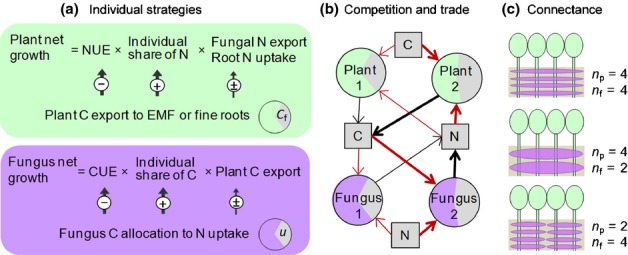
(a) An individual's strategy, that is, plant carbon (C) export (*C*_f_) to ectomycorrhizal fungi (EMF) and fungal allocation to nitrogen (N) uptake (*u*), is optimized to maximize net growth (including reproductive production) via three factors: its hosts' total resource export (and/or nonmycorrhizal N uptake by plants), resource share obtained in intra-party competition, and resource use efficiency (N use efficiency (NUE) or C use efficiency (CUE)). (b) Competition is illustrated by uptake (red arrows) and export (black arrows) of C and N. Arrow thickness indicates flux rates: plant 2 exports more C to EMF, which results in a higher N uptake than in plant 1. Fungus 2 delivers more N and a higher N : C exchange rate, and thus receives more C than fungus 1. (c) Each plant hosts multiple competing fungi (the number of fungal partners per plant host (*n*_f_)) and each fungus hosts multiple competing plants (the number of plant partners per fungal host (*n*_p_)). See Supporting Information Methods S1 for the full mathematical description of the model.

We developed a mycorrhizal symbiosis model (Fig.1, Supporting Information Methods S1, Table S1, Fig. S1) based on established equations for N-limited plant growth (Franklin *et al*., 2012) and C-limited (or C- and N-co-limited) fungal growth (Näsholm *et al*., 2013). However, in contrast to previous stand-based applications of these physiological models, here each fungus and plant is allowed to individually adjust its strategy in terms of internal C–N allocation and interaction (trading) with its (multiple) symbiotic partners. The interaction among multiple individuals is modeled as a market, where each plant supplies C in exchange for N and each fungus supplies N in exchange for C. The resulting N : C exchange rate is analogous to the price in a financial market and will depend on the supply and demand of C and N, the intensity of market competition (e.g. competition for plant C among multiple N-supplying fungi), and an individual's influence on supply (e.g. one fungus' N export influence on plant C supply). A mycorrhizal network provides the physical structure facilitating these market interactions.

### Individual physiology

Net plant growth is modeled as photosynthesis minus C costs of respiration, litter production, and C export to EMF, where both photosynthesis and respiration are functions of plant N content. Photosynthesis is also a function of light absorption and atmospheric CO_2_, where elevated CO_2_ enhances photosynthetic N use efficiency (Franklin, 2007). Plants regulate C export to EMF depending on their N demand and the fungal N return. Gross N uptake from soil is a function of soil N availability and the N uptake component of EMF biomass, or fine-root biomass for nonmycorrhizal roots, limited by its soil exploration efficiency at low soil N, and by uptake capacity at high N. Nitrogen uptake by EMF that is in excess of their N demand for growth (determined by C import times a fixed biomass N : C ratio) is exported to host plants (Näsholm *et al*., 2013).

**Figure 2:**
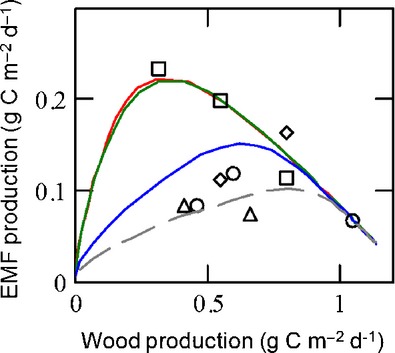
Modeled (lines) versus measured (symbols) production (biomass growth) of ectomycorrhizal fungi and wood along soil nitrogen (N) gradients in boreal forests (Nilsson *et al*., 2005; Ekblad *et al.,* 2013; Methods S1), where the sites are Varjisån (squares), Flakastugan (diamonds), Betsele (circles), and Kryddgrovan (triangles). Line colors correspond to connectance scenarios, that is, numbers of fungal partners per plant and vice versa (*n*_f_ and *n*_p_, respectively): green (*n*_f_ = 8; *n*_p_ = 8), red (*n*_f_ = 2; *n*_p_ = 8) blue (*n*_f_ = 8; *n*_p_ = 2), and gray dashed (*n*_f_ = 100; *n*_p_ = 1).

Physiological parameter values were estimated based on our recent dual stable C and N isotope labeling experiments in boreal forest (Högberg *et al*., 2010; Näsholm *et al*., 2013) and other published data typical of boreal pine forests; for example, the biomass N : C ratio was estimated to be 0.03 for foliage and fine roots (Luoma, 1997) and 0.085 for EMF (Mikusinska *et al*., 2013), the mean life-span of foliage was estimated to be 4 yr, that of fine roots 2 yr (Keel *et al*., 2012), and that of EMF 17 d (Högberg *et al*., 2008, 2010), and light-saturated photosynthetic N use efficiency was estimated to be 0.14 g C g N^−1^ h^−1^ (Luoma, 1997). Unknown parameters were adjusted to match the range of measured EMF and tree productivity in soil N gradients in boreal forests (Fig.2; Methods S1).

### Strategies, trade, and competition

Importantly, the model captures two critical features of ectomycorrhizal symbiosis not addressed in existing models: (1) each plant interacts simultaneously with multiple EMF and each fungus interacts with multiple plants (i.e. a mycorrhizal network; e.g. Southworth *et al*., 2005; Beiler *et al*., 2010), and (2) the strategies of individuals adapt to the environment (biotic and abiotic) by maximizing fitness, approximated by net growth (including reproduction) for plants (Franklin *et al*., 2012) and reproductive growth for fungi (Pringle & Taylor, 2002). A plant's strategy in the model is defined by its C allocation to EMF (*C*_f_), which in effect regulates the trade-off between C investments in N uptake (via EMF) and C use for growth. A fungus strategy is defined by its ratio (*u*) of C allocation to reproductive growth versus N uptake components, in line with the observed trade-off between intrinsic growth rate and potential N uptake capacity (measured as efficiency of protein mineralization) among EMF species (Eaton & Ayres, 2002). Changes in *u* may reflect both phenotypic plasticity and changes in active EMF community (species) composition. Thus, we do not differentiate between acclimation of *u* of the existing EMF and replacement of these EMF with new individuals of a different (or the same) species that have a different *u*. The responses of other characteristics of the fungal community to soil N availability (e.g. uptake capacity for different forms of N and spatial distribution) are implicitly subsumed in the response of N uptake to N availability (Methods S1).

As a result of the resource trading, the strategy of an individual (*u* or *C*_f_) affects not only the individual's own nutrient status and fitness but also those of its trading partners. For example, if a fungus increases N export (via increased *u*) this may reduce or increase the C export by its plant partners, that is, change the plants' strategy, which implies that the strategies of plants and EMF are always linked. However, if each fungus has more than one plant partner, its influence on the plant strategies, that is, C export, is ‘diluted’ in proportion to how many fungal partners each plant has, and vice versa for a plant's influence on its fungal partners. Thus, in the presence of multiple partners, a fungus' potential to influence its host plants' C export declines; instead, competition for this C with the other fungal partners of the same plants becomes important. If one fungus returns more N per additional plant C received than the other fungal partners, the plant can increase its fitness by trading more with this particular partner. Thus, we assume that fungal partners of the same host plants compete via their N : C exchange rate, that is, N exported to the plants per C received (and vice versa for a plant competing for fungal N export). The relative fraction of plant-derived C obtained by an individual fungus (*F*_*i*_) is a function of its N : C exchange ratio (*x*) with its host plants relative to its competitors, 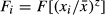, where 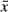 is mean *x* among all competitors*,* and *z* determines how strongly *F*_*i*_ is affected by *x*_*i*_ (partner discrimination). In the absence of an empirical basis for estimating *z* we used a value of 1 and tested the effect of higher and lower values. In contrast to the proximity of different fungal partners on plant roots, the spatial separation between different plant partners of a fungus should enable strong partner discrimination, that is, a high *z*, in line with empirical observations of AMF (Lekberg *et al*., 2010). Strong partner discrimination will force equal N : C exchange ratios among competing plants so that they effectively compete for fungal N via how much fungal biomass they support. Thus, the fraction of fungal N export a plant receives in competition with other plant partners of the same EMF is a function of its C export (*C*_f_) relative to its competitors.

An optimal, or equilibrium, strategy (evolutionarily stable strategy) means that no individual can gain fitness by changing its strategy, which corresponds to resource exchange with equal marginal return from all trading partners. Under our assumption of identical individuals in each party, this principle corresponds to the reciprocal rewards concept (Kiers *et al*., 2011) but it is more general because it does not exclude nonreciprocity if partners of the same host differ in their C or N supply versus demand (price) response, for example for different plant species linked to the same mycorrhizal fungus (Walder *et al*., 2012).

An additional constraint on the N : C exchange ratio is set by the possibility for plants to switch to a nonmycorrhizal strategy. If the market mechanisms described above result in an N : C exchange ratio that renders the nonmycorrhizal roots more profitable for a plant than mycorrhizal roots, to persist, EMF must increase their N : C exchange ratio via increased allocation to N uptake (*u*) so that their utility for plants matches that of nonmycorrhizal roots. EMF with lower *u* will not receive C from the plants.

### Symbiotic stability

The ecological stability of the mycorrhizal plant strategy depends on its competitiveness against a nonmycorrhizal strategy, which in turn depends on soil N availability. EMF have a much higher (by a factor of 300 in the model) specific N uptake efficiency than nonmycorrhizal roots at low soil N availability (Jennings, 1995; Smith & Read, 2008), whereas the efficiencies converge for saturating N availability (Methods S1). Thus, under increasing soil N availability, the mycorrhizal advantage in gross N uptake rate declines and is eventually outweighed by the C costs and fungal N immobilization it incurs. As a consequence of the differences in N uptake and resulting soil N depletion between mycorrhizal and nonmycorrhizal roots, the relative benefits of each root strategy also depend on the strategy of the surrounding competitors of the plant (Methods S1). In essence, the nonmycorrhizal strategy can invade if a plant can gain fitness by growing nonmycorrhizal roots in a population of mycorrhizal plants and vice versa. If both strategies can invade each other they will coexist.

## Results

### Results and evaluation against empirical observations

**Figure 3:**
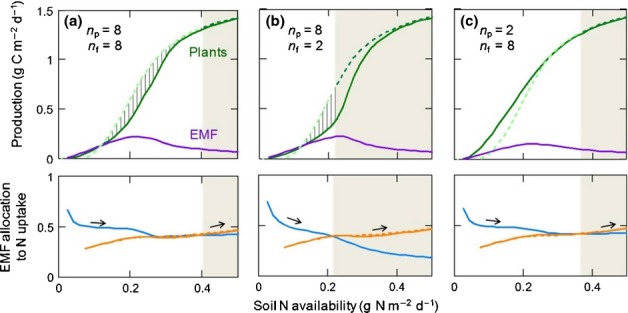
Modeled net growth of plants with and without mycorrhiza (solid and dashed green lines, respectively) and ectomycorrhizal fungi (EMF; purple lines), where in (a) each plant has eight fungal partners (*n*_f_ = 8) and each fungus has eight plant partners (*n*_p_ = 8), in (b) each plant has fewer fungal partners (*n*_f_ = 2; *n*_p_ = 8), and in (c) each fungus has fewer plant partners (*n*_f_ = 8; *n*_p_ = 2). Fungal strategy, that is, fractional carbon (C) allocation to nitrogen (N) uptake components (*u*; lower panels), is determined either by inter-fungal competition (nonshaded area, blue line) or by competition with nonmycorrhizal roots (shaded area), which determines the minimum *u* at which a mycorrhizal plant strategy can invade a nonmycorrhizal strategy (dashed orange line) and avoid invasion of a nonmycorrhizal strategy (solid orange line). The mycorrhizal strategy may persist although it reduces plant growth (vertically dashed area) compared with a nonmycorrhizal strategy (light-green dashed line).

**Figure 4:**
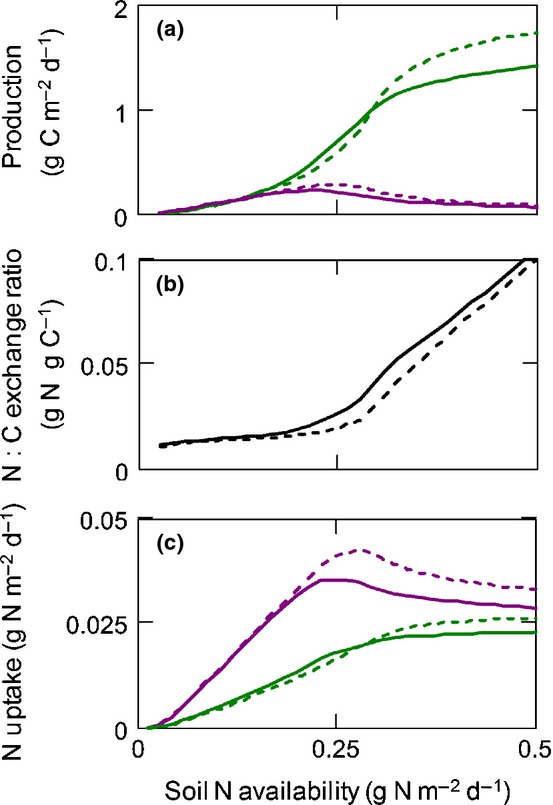
Model results under ambient CO_2_ (solid lines) and elevated CO_2_ (dashed lines). (a) Plant net growth (green line) and growth of ectomycorrhizal fungi (EMF; purple line). (b) Plant–fungal nitrogen : carbon (N : C) exchange ratio. (c) N uptake of EMF (purple lines) and N transfer to plants (green lines). Plant-fungi connectance corresponds to Fig. 3(a), i.e. each plant had eight fungal partners (*n*_f_ = 8) and vice versa (*n*_p_ = 8)

We evaluated the model's predictions in comparison to published empirical results on effects of soil N availability and atmospheric CO_2_ in boreal or temperate ectomycorrhizal forests. The modeled responses of plants and EMF to soil N are modulated by the number of fungal partners per plant host (*n*_f_) and the number of plant partners per fungal host (*n*_p_; Fig.3), which determines the importance of intra-party competition for an individual's strategy (Fig.1). Competition induces tragedy-of-the-commons effects in each party that prevent either the plants or the EMF from collectively optimizing the N : C exchange rate to maximize their benefits. A hypothetical absence of inter-fungal competition (a single fungus individual monopoly) would quickly make plants abandon the fully mycorrhizal strategy as N availability increases or, without a nonmycorrhizal option for the plants, would severely hamper plant productivity (Fig. S2). However, as long as plants have a nonmycorrhizal option or there is inter-fungal competition for plant C, C and N fluxes between plants and EMF will largely follow the basic principles of supply and demand versus cost (here the N : C exchange rate). As the N demand of fungi is constrained by their access to C from the plants, increasing soil N availability leads to higher N export to plants, resulting in increased plant growth (Fig.3) and higher N : C exchange rate (Fig.4), in line with results from field experiments (Näsholm *et al*., 2013). At the same time, plant N demand drives C allocation to EMF to first increase and then decline as other factors (e.g. light) constrain plant N demand (Fig.3), explaining observed patterns of EMF growth (Nilsson *et al*., 2005; Hasselquist *et al*., 2012; Kjøller *et al*., 2012) analogously to fine-root allocation in nonmycorrhizal plants (Franklin *et al*., 2012). Although often only the declining phase of mycorrhizal response to increasing N availability is observed, it must be preceded by an increasing phase as productivity of both plants and hosted EMF must converge to zero at zero N availability. In response to elevated atmospheric CO_2_, the plant–fungal N : C exchange rate is reduced (Fig.4b) in line with observations (Alberton & Kuyper, 2009). N transfer to plants and corresponding plant net growth (mainly stem growth in trees) are enhanced by elevated CO_2_ at high soil N availability but not at low soil N availability (Fig.4b,c) in agreement with stand-scale observations (Oren *et al*., 2001; Dieleman *et al*., 2010).

As a consequence of the intra-party competition effect, a fungus' N export to its host plant (and plant growth) increases with the effective number of competing fungal partners (*n*_f_) while plant C export to a fungus increases with the number of plant partners per fungus (*n*_p_) (Fig.3). Supporting this result, plants with several fungal partner species increased N uptake and growth at elevated CO_2_ more than plants with only one fungal partner (Alberton *et al*., 2005). Although not modeled, an additional partner may also have a competitive advantage that drives improved N : C exchange rate (for the host) in competition with existing partners. Thus, both fungi and plants should strive to increase the number of partners, which means that connectance should increase concurrently for plants and EMF, until other factors (e.g. grazing; Högberg *et al*., 2010) limit further network expansion. Increased connectance enhances plant C allocation to EMF growth and shifts the strategy of the EMF toward increased N uptake (Fig.3). Both these effects increase gross N uptake by EMF, which depletes marginal soil N availability and competitively hampers a nonmycorrhizal strategy. Importantly, this can stabilize the mycorrhizal strategy even when it reduces plant growth compared with a nonmycorrhizal strategy. Thus, while the mycorrhizal strategy allows plants to survive at lower soil N than without mycorrhiza, the symbiosis may reduce plant growth but still persist at higher soil N availability (Fig.3).

**Figure 5:**
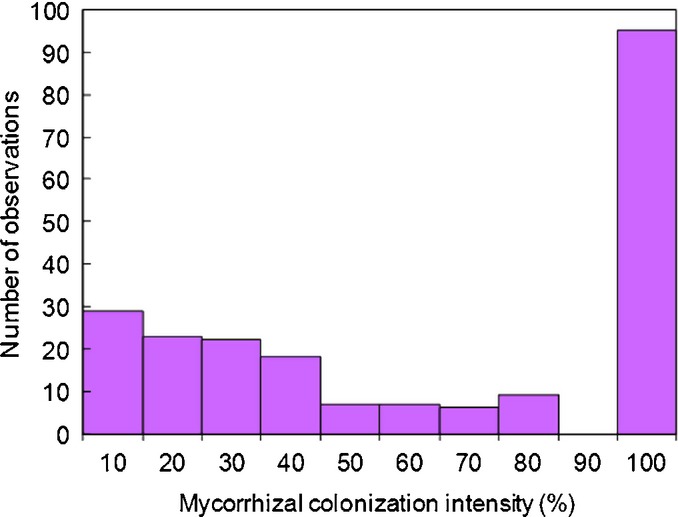
Field measurements of ectomycorrhizal colonization intensity, for 125 species and 44 sites in the former Soviet Union (Akhmetzhanova *et al*., 2012), show high frequency of observations at very high or relatively low colonization intensity and fewer observations at intermediate colonization intensity, as predicted by our model. Statistical testing by *k*-means cluster analysis separated two clusters centered at high (95%) and low (21%) values with *P *<* *0.0001. The colonization intensity is measured in per cent of maximal colonization intensity, based on the mean number of mycorrhizal root tips per root length for each species at each site, relative to a reference scale (Akhmetzhanova *et al*., 2012).

When soil N availability increases to a level where a nonmycorrhizal strategy can invade, EMF only persist if their utility for a plant matches that of nonmycorrhizal roots, which requires EMF to invest more in N uptake (increase *u*) than in the absence of a nonmycorrhizal strategy (Fig.3 versus Fig. S2). However, EMF will not increase *u* more than necessary as this would reduce fitness. For a plant this means a switch from a clear advantage of the mycorrhizal strategy to equal utility of mycorrhizal and nonmycorrhizal roots. At the same time, there exists a *u* that allows the mycorrhizal strategy to invade a resident nonmycorrhizal strategy. Thus, both root strategies may coexist under further increasing soil N availability until EMF are forced to allocate all resources to N uptake (*u*→1, leaving nothing for reproductive growth), making the symbiosis unviable. Although mutual invasibility and convergence of the utilities of the alternative root strategies do not imply strictly equal frequency, they suggest that, on average, mycorrhizal and nonmycorrhizal roots coexist in similar proportions. These results agree with the patterns typically observed in gradients of increasing soil N availability: while plant growth gradually increases there is almost complete mycorrhizal root colonization under a range of low soil N availability, followed by coexistence of nonmycorrhizal and mycorrhizal roots, and finally disappearance of EMF (Taylor *et al*., 2000; Högberg *et al*., 2003; Nilsson *et al*., 2005; Kjøller *et al*., 2012). A corresponding negative trend was also found for soil mycelia where ectomycorrhizal species declined and disappeared (Högberg *et al*., 2014). Furthermore, because the increasing fungal allocation to N uptake components occurs at the expense of reproductive allocation (increased *u*), it agrees with empirically observed reductions in fruiting-body (reproductive structures) production at high N additions (Peter *et al*., 2001; Hasselquist *et al*., 2012). Although many factors influence at which point the plants switch between a pure mycorrhizal strategy and a mixed strategy with both mycorrhizal and nonmycorrhizal roots, the model implies that along environmental gradients there should always be abrupt shifts between very high (≈ 100%) mycorrhizal colonization and much lower colonization. Although not supported by all studies (e.g. Børja & Nilsen, 2009) this threshold behavior agrees with observations of ectomycorrhizal colonization intensity across a wide-ranging data set comprising 125 species and 44 sites (Akhmetzhanova *et al*., 2012), which were clustered at very high or relatively low values (Fig.5).

## Discussion

### Scope and limitations

We have shown that market mechanisms can shape the strategies of plants and mycorrhizal fungi to stabilize the mycorrhizal symbiosis under a wide range of soil N availability. Whereas the model formulation is general enough to encompass ectomycorrhizal symbiosis as well as arbuscular mycorrhizal (AM) symbiosis, we have chosen to focus the model evaluation on N-limited ectomycorrhizal forest. The main reason for this focus is the dominant role that the symbiosis plays in these ecosystems in terms of N and C cycling (e.g. Näsholm *et al*., 2013). Hypothetically, many of our results should be qualitatively valid also for AM symbiosis under nutrient (N or phosphorus)-limited conditions. However, the more open N cycle (less N limitation) in forests with predominantly AM symbiosis (Phillips *et al*., 2013) suggests that the stabilization of symbiosis via nutrient depletion feedback (see ‘Implication 2: mutual stabilization of nitrogen limitation and ectomycorrhizal symbiosis’) is less relevant in AM-dominated ecosystems.

Another difference between mycorrhizal types is that significant amounts of organic N are taken up by EMF in ectomycorrhizal forests whereas inorganic N uptake dominates in AMF (Phillips *et al*., 2013). A change from an organic to an inorganic N source would increase the N : C ratio of fungal uptake and metabolic pools and would therefore lead to a higher N : C exchange ratio with the plants in our model. This would lead to quantitative shifts in our results and slightly different values of the fitted parameters (uptake capacities) but no qualitative changes in the conclusions.

### Implication 1: two stability regimes of ectomycorrhizal symbiosis

The model suggests a previously not recognized dichotomy in how the strategies of EMF respond to soil N availability: (1) partner discrimination by plants induces competition for C among their fungal partners that enhances fungal C allocation to N uptake, stabilizing a fungal strategy beneficial for host plants at low N availability, whereas (2) competition (or choice) between mycorrhizal and nonmycorrhizal plant strategies constrains fungal strategy at higher N availability. However, although empirical evidence supports the general importance of competition in structuring EMF communities (Kennedy, 2010), the specific mechanism (1) has been empirically confirmed only for AM symbiosis (Kiers *et al*., 2011; Fellbaum *et al*., 2012). Without mechanism (1), that is, plant discrimination among partner fungi, there would be no stabilizing effect of inter-fungal competition at low N availability and the stability of the symbiosis would rely solely on mechanism (2) (*z *=* *0 in Fig. S3). The model would (with some parameter adjustments) still be able to reproduce many observed phenomena, including the effects of soil N availability and elevated CO_2_ on N : C exchange rates (Alberton & Kuyper, 2009; Näsholm *et al*., 2013) and the stronger decline in fruiting-body production than in mycelia of EMF under high N availability (Wallenda & Kottke, 1998; Peter *et al*., 2001; Hasselquist *et al*., 2012). However, the necessity of both mechanisms (1) and (2) is supported by the high colonization rates of EMF at low soil N, and the threshold behavior of ectomycorrhizal colonization intensity (Fig.5), which indicates switching between the two controlling mechanisms. Mechanism (2) alone, that is, absence of intra-party fungal competition, would lead to a continuously declining fungal allocation to N uptake components at decreasing soil N availability, maintaining similar utility of mycorrhizal and nonmycorrhizal plant strategies and thus lower ectomycorrhizal colonization at low N availability than the near 100% commonly observed (Taylor *et al*., 2000; Fig.5).

Under the ongoing global rise in N deposition, switching between the two mechanisms delineated above may influence not only the colonization rate and productivity of EMF, but also forest N cycling. While forests growing at high and very low N availabilities should experience gradual change in response to N deposition, trees growing at intermediate N availability may suddenly reduce mycorrhizal colonization (as a result of switching of stabilizing mechanisms from (1) to (2)), which reduces N retention and may induce ecosystem N leaching (Bahr *et al*., 2013; Högberg *et al*., 2013).

### Implication 2: mutual stabilization of nitrogen limitation and ectomycorrhizal symbiosis

**Figure 6:**
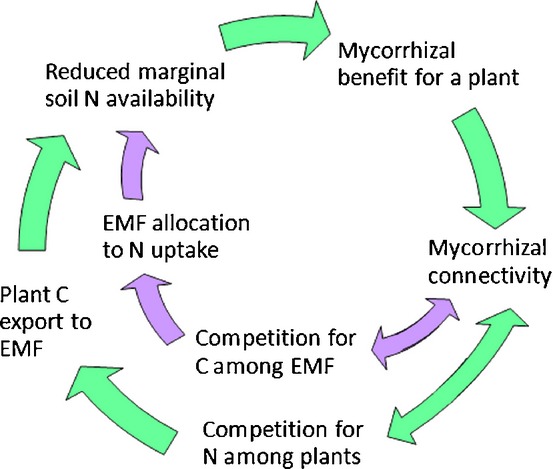
Feedback loop stabilizing the mycorrhizal strategy, showing the interrelation between effects of ectomycorrhizal fungi (EMF; purple arrows) and plants (green arrows). Increased fungal–plant (mycorrhizal) connectivity enhances competition among both EMF and plants, which drives plants to increase carbon (C) export to EMF and EMF to increase nitrogen (N) uptake and N export to plants. These effects interact in depleting available soil N, which favors the mycorrhizal strategy compared with a nonmycorrhizal plant strategy, which in turn promotes mycorrhizal connectivity.

In addition to the primary mechanisms (1) and (2), the model postulates that mechanism (1) gives rise to a secondary stabilizing mechanism: stabilization of the mycorrhizal strategy via reduction of marginal N availability (N uptake gain per additional root mass), that is, environmental resource feedback (Fig.6). Thus, the mycorrhizal symbiosis creates its own environment where it outcompetes nonmycorrhizal plant strategies. Its potential to stabilize mycorrhizal symbiosis even when it reduces plant growth suggests that it may be responsible for the large fraction of negative effects among observations of mycorrhizal effects on plant growth (Corrêa *et al*., 2008, 2012). Importantly, the feedback stabilization of N limitation and mycorrhizal symbiosis under low N availability suggests that it underpins the syndrome of low productivity and mycorrhizal dominance in boreal forests (Högberg *et al*., 2011; Näsholm *et al*., 2013). This feedback effect would be exacerbated if EMF contribute to continuing soil N immobilization, as suggested by the increase in natural abundance of ^15^N with increasing soil depth observed in ectomycorrhizal forests (Högberg *et al*., 1996; Billings & Richter, 2006; Hobbie & Ouimette, 2009). Although the full extent of the feedback stabilization hypothesis is not easily tested experimentally, the link between high N retention and EMF has been established in plots recovering from long-term N addition (Högberg *et al*., 2011, 2014).

### Implication 3: ectomycorrhizal symbiosis hampers CO_2_ fertilization of tree growth

The model suggest that N transfer to plants and resulting plant net growth (growth minus litter production) is enhanced by elevated CO_2_ at high soil N availability but not at low N availability, where it may even decrease (Fig.4). Thus, only at sufficiently high N availability will trees in ectomycorrhizal forests increase stem growth under rising CO_2_. This suggests that mycorrhizal symbiosis may make the strongly N-limited boreal forests particularly susceptible to progressive N limitation postulated as a consequence of rising atmospheric CO_2_ (Luo *et al*., 2004; Alberton *et al*., 2007). The underlying reason for this is that, at low soil N availability, the increase in tree C export to EMF in response to elevated CO_2_ enhances N immobilization in EMF biomass more than N export to trees, which is reflected in a reduced N : C exchange rate (Fig.4b). Thus, the trees generate a self-imposed N limitation by excessive C export to EMF in response to elevated CO_2_. Although this may seem highly maladaptive, from an individual tree's perspective it is an adaptive response (increasing fitness) to competition for N among trees. Just like in the stabilization of a nonbeneficial mycorrhizal plant strategy discussed above (Implication 2: mutual stabilization of nitrogen limitation and ectomycorrhizal symbiosis), individual fitness maximization leads to a collective loss, that is, a tragedy-of-the-commons effect.

### Theoretical implications in a wider context: intra-party competition stabilizes symbiosis for better or worse

The importance of intra-party competition in a market context (out-bidding) for the stability of mutualism has long been recognized (Noë & Hammerstein, 1994; Jones *et al*., 2012; Werner *et al*., 2014). This principle has been applied to mycorrhizal symbiosis, showing how a single plant should optimize the use of multiple fungal partners (Kummel & Salant, 2006). Our model extends this theory by accounting for adaptive strategies in both parties of the symbiosis, quantifying the effects of partner numbers, and exploring its potential to stabilize nonmutualistic symbiosis. Because the beneficial effect for a host of intra-party competition among its partners increases with the number of partners (Fig.3), this mechanism may drive the observed positive relationship between connectance and stability in mutualistic networks; that is, a species’ chance of survival increases with the number of partners (James *et al*., 2012). Similarly, the mechanism may contribute to the observed increase in productivity with diversity of mycorrhizal fungi (van der Heijden *et al*., 1998), because species diversity should correlate with the number of individuals and the range of fungal strategies that compete for plant C. However, while mycorrhizal networks have been viewed as a facilitating ‘socialism’ in terms of nutrient sharing among plants (Van Der Heijden & Horton, 2009), in our theory they rather promote ‘capitalism’, by enhancing competition among profit-maximizing trading individuals. For example, when different plants share a fungal partner delivering N, the allocation of N to each plant is driven by the fungus' strategy to maximize C return rather than by a plant behavior to share N with other plants. Thus, perhaps expectedly, the resulting increased trading is not always beneficial for both parties of the trade. Whereas partner competition enhances resource transfer to a host, our model shows that the resulting increase in resource depletion can stabilize mycorrhizal symbiosis even if a nonmycorrhizal strategy would have been more productive for the plants. More generally, this implies that resource feedback can drive inherently mutualistic (trading) entities into nonmutualistic cooperation (Figs3a,b, 6). This resource feedback stabilization of a suboptimal strategy has interesting parallels in other market contexts, such as lock-in of inferior energy technologies (Unruh, 2000), highlighting the cross-disciplinary relevance of such mechanisms.

### Conclusions and way forward

While recent theoretical progress on mycorrhizal symbiosis mainly has focused on the evolution and stability of mutualism in pair-wise interactions, empirical research has revealed a picture of multiple simultaneous and highly dynamic interactions. Here we have taken a step to extend theory in this direction, based on a model that links the strategy (governed by evolutionary principles) of each individual to its multiple exchangeable trading partners and competitors in a network.

The model is able to reproduce a wide range of observed responses to N and atmospheric CO_2_ in ectomycorrhizal forest, indicating that it captures relevant mechanisms despite its highly simplified representations of ecophysiology and functional diversity of EMF. However, more empirical research is needed to allow further evaluation of the hypotheses generated and some of the model's assumptions. A central assumption is that a host is able to discriminate and induce competition among its partners, which benefits the host and promotes symbiotic stability. While such partner discrimination has been confirmed for AM symbiosis, empirical quantification and investigation of its role in ectomycorrhizal symbiosis are urgently needed. Furthermore, while plant allocation responses to resource availability are relatively well known, the importance of corresponding acclimation/adaptation of fungal strategies indicated by our results is much less explored. Progress in understanding and modeling of mycorrhizal ecosystems would strongly benefit from exploration of additional traits and trade-offs delineating functional/strategic diversity in EMF, including, for example, life-span, biomass N : C ratio, and enzyme production.

The results suggest that persistence of ectomycorrhizal symbiosis in boreal forests emerges at the ecosystem level, via positive feedback between low nutrient availability and stability of the mycorrhizal plant strategy. This mechanism may also have the capacity to eliminate the expected positive effect of rising CO_2_ on tree growth or even reverse it to a negative effect in strongly N-limited boreal forests. The potential significance of these results for the global C cycle underlines the need for an ecosystem perspective in understanding mycorrhizal symbiosis and the importance of including the effects of mycorrhizal symbiosis in applied forest and vegetation models.

## Supporting Information

**Fig. S1** Comparison of nitrogen uptake for roots and mycorrhizal fungi. **Fig. S2** Modeled effects of mycorrhizal symbiosis on trees and mycorrhizal fungi when trees lack the option of a nonmycorrhizal strategy. **Fig. S3** Modeled effects of variation in the fungal ability to discriminate among plant partners (parameter *z*) on productivity and symbiotic stability in the forest. **Table S1** Model parameters and variables **Methods S1** Full model description.
