# Supplementary material for: Forests trapped in nitrogen limitation – an ecological market perspective on ectomycorrhizal symbiosis
Source: New Phytol. 2014 May 14;203(2):657–66. doi: 10.1111/nph.12840 (PMC4199275; doi:10.1111/nph.12840)
Supplement: Supplementary file 1 — Fig. S1 Comparison of nitrogen uptake for roots and mycorrhizal fungi. Fig. S2 Modeled effects of mycorrhizal symbiosis on trees and mycorrhizal fungi when trees lack the option of a nonmycorrhizal strategy. Fig. S3 Modeled effects of variation in the fungal ability to discriminate among plant partners (parameter z) on productivity and symbiotic stability in the forest. Table S1 Model parameters and variables Methods S1 Full model description. [file nph0203-0657-sd1.pdf]

Supporting Information Methods S1, Table S1, Figs S1–S3

Methods S1: Mathematical model description

Table S1. Model variables and parameters.

Symbol	Value	Unit	Description
			dependent variables
B_r, B_f, B_{fu}		gC m^{-2}	Biomass of fine-roots, EMF, and EMF N uptake components
C_r, C_f		gC m^{-2} d^{-1}	C use for fine-root growth, and C export to EMF
F		-	Fraction resource obtained by one individual among competitors
G		gC m^{-2} d^{-1}	Net plant growth (GPP – respiration – litter production)
G_f		gC m^{-2} d^{-1}	Net EMF reproductive production
N_c, N_r		gN m^{-2} d^{-1}	N in canopy and fine-roots
N_p		gN m^{-2}	Plant N uptake
P		gC m^{-2} d^{-1}	Net canopy C uptake (photosynthesis)
P_f		gC m^{-2} d^{-1}	EMF biomass production
U		gN m^{-2} d^{-1}	N uptake by roots or hyphae
U_c		gN m^{-2} d^{-1}	N uptake capacity of fine-roots or EMF
u		-	EMF biomass fraction contributing to N uptake
W		gC m^{-2} d^{-1}	Plant C costs of respiration and litter production
x		gN gC^{-1}	EMF-Plant N:C exchange rate
			independent variables and parameters
a	0.14	gC gN^{-1} h^{-1}	Light saturated photosynthesis per canopy N (Luoma, 1997)
c_c, c_r, c_G, c_f	0.03, 0.03, 0.01, 0.085	gN gC^{-1}	N:C ratio of foliage (Luoma, 1997), fine-roots, plant net growth, and EMF (Mikusinska *et al.,* 2013)
d	0.25	m	Effective soil depth
e_u	0.5, 0.0017	m^3 gC^{-1}	Soil volume explored per EMF uptake components and fine-root biomass, respectively[#]
f_r	1	-	Minimum fine-root production per growth of EMF (Hasselquist *et al.,* 2012)
N_{av}		gN m^{-3}d^{-1}	Soil N availability = maximal N uptake per soil volume
h	12	h	day length in hours
n_p, n_f		-	Number of competitors for plants and EMF, respectively. Examples of observed values range from 1 -20 (Southworth *et al.*, 2005; Beiler *et al.*, 2010)
Q	1.5	gC h^{-1}	N saturated photosynthetic capacity = photosynthetic quantum efficiency (Wong *et al.*, 1979) times photosynthetically active radiation.
t_c, t_r, t_f	800, 400, 17	d	Lifespan of foliage[*], fine-roots (Keel *et al.,* 2012), and EMF (Högberg *et al.,* 2008; Högberg *et al.,* 2010), respectively
u_{cB}	0.1	gN gC^{-1} d^{-1}	N saturated N uptake capacity per root or EMF biomass[#]
w	0.25	gC gN^{-1} d^{-1}	Plant litter and respiration C costs per canopy N[#]
y_f	0.3	gC gC^{-1}	Fungal net C use efficiency (Sinsabaugh *et al.*, 2013)
Y_p	0.7	gC gC^{-1}	Plant growth C conversion efficiency (Choudhury, 2001)
z			Exponent of the partner discrimination relationship

[#] calibrated to match measured data (see below- Model parameterization, and Fig. 2)
[*]typical values in boreal pine forest were used. All rate values refer to the growing season (e.g. *d* means growing season days) spanning 150 days.

Nitrogen uptake

Gross N uptake (both for fine-roots and ectomycorrhizal fungi (EMF); U; eq. 1) is determined by maximal (N saturated) uptake capacity of the whole root system or EMF population (U_c, eq. 2), soil N availability (N_{av}) and soil depth (d).

$$U = \frac{U_c\, N_{av}\, d}{U_c + N_{av}\, d} \tag{1}$$

U_c in turn depends on the biomass (B) of fine-roots or fungal uptake components, the specific maximal N uptake capacities (u_{cB}) and the specific maximum N uptake efficiency at low soil N availability (e_u), which is equivalent to the soil volume accessed per B.

$$U_c = B\frac{u_{cB}\, N_{av}\, e_u}{u_{cB} + N_{av}\, e_u} \tag{2}$$

In terms of N uptake, fine-roots and hyphae of EMF differ only in their specific N uptake efficiency (e_u, eq. 2), which is much higher for hyphae than fine-roots. This difference combined with the higher turnover (shorter life-span) of EMF compared to fine-roots mean that mycorrhizal N uptake is more cost efficient at low than at high soil N availability (Fig. S1).

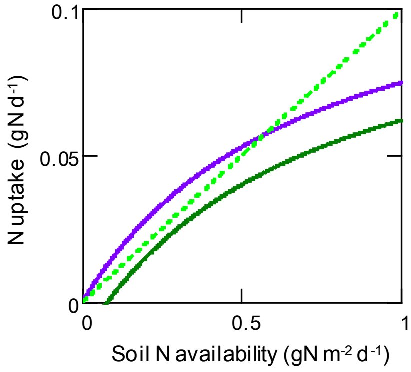

Figure. S1. Modeled nitrogen (N) uptake by ectomycorrhizal fungi (EMF, solid purple line), N transfer to mycorrhizal plants (solid green line), and N uptake of a non-mycorrhizal plant (dashed green line) for an equal fixed amount of C (= 0.5 g m^{-2} d^{-1}) invested in the root system.

Plant physiology

To model the interaction between plants and EMF, plant growth and allocation is expressed in terms of carbon export to EMF (C_f) and N uptake (N_p) via EMF or directly from soil. C_f is optimized to maximize net growth (G_p, including biomass increase and reproductive production), which is a reasonable fitness proxy (Franklin *et al.,* 2012). We calculate G_p based on a C flux balance equation (eq. 3) expressed in terms of photosynthesis (P, eq. 4) minus C costs due to maintenance respiration and litter production (W, eq. 5), and C_f. Both P and W are functions of N, which links the C balance equations to the N balance equation (eq. 6). This model corresponds to the forest stand model developed in (Franklin *et al.,* 2012) with the addition of C export to EMF.

$$G_p = y_p\left(P - W - C_f\right) \tag{3}$$

$$P = h\frac{a\,N_c\,Q}{a\,N_c + Q} \quad (4)$$

$$W = w\left(N_c + N_r\right) \quad (5)$$

$$N_p = \frac{N_c}{t_c} + \frac{N_r}{t_r} + c_G\,G_p \quad (6)$$

$$N_r = B_r\,c_r\,,\ \text{where}\ B_r = B_{r0} + B_{rf} \quad (7)$$

$$B_{r0} = C_{r0}\,t_r \quad (8)$$

$$B_{rf} = f_r\,C_f\,y_f\,t_f \quad (9)$$

Eq. 4 means that P is limited by radiation (Q= photosynthetically active radiation × photosynthetic quantum efficiency), canopy N (N_c) and day-length (h). The parameter a is the light saturated photosynthetic capacity per leaf N. In eq.5, w is respiration and litter production per N in foliage (N_c) and fine-roots (N_r). In eq. 6, N_p is plant N uptake, t_c and t_r are life-spans of foliage and fine-roots, and c_G is the N:C ratio of net growth. In eq. 7, B_r is the biomass of fine-roots, which includes non-mycorrhizal roots (B_{r0}, eq. 8) and mycorrhizal roots (B_{rf}, eq. 9).We assume that B_{rf} is a constant factor (f_r) of the hosted fungal growth, which in turn is a function of C export to EMF and their growth C efficiency, y_f.

Using eqs. 3-9, G_p can be expressed analytically (expression too large to show here) in terms of fixed parameters and the variables nitrogen uptake (N_p), C export to EMF (C_f), and C export to non-mycorrhizal roots (C_{r0}) (eq. 10).

$$G_p = f[C_f, C_{r0}, N_p] \quad (10)$$

Fungal physiology

Fungal biomass production (P_f, eq. 11) is modeled as a function of plant C export (C_f) as done in (Näsholm *et al.,* 2013), with the addition that fungal biomass (B_f) is divided into components for N uptake (B_{fu}) and reproductive growth, which is produced at a rate G_f,(eq. 11). The fraction of uptake components (u) relative to reproductive growth (1-u) defines the strategy of a fungus.

$$G_f = P_f\,(1-u)\,,\ \text{where}\ P_f = y_f\,C_f \quad (11)$$

$$B_{fu} = B_f\,u\,,\ \text{where}\ B_f = P_f\,t_f \quad (12)$$

In eq. 12, t_f is the life-span of EMF.

EMF take up N as a function of B_{fu} ($U[B_{fu}]$, eq. 13) of which a part is used for their own growth and the rest (N_p) is exported to the plants (Näsholm *et al.,* 2013).

$$N_p = U[B_{fu}] - P_f\,c_f \quad (13)$$

In eq. 13, c_f is fungal biomass N:C ratio.

The representation of fungal growth in terms of only two components was made to focus on the most relevant strategic component of fungal adaptation to N availability (Eaton & Ayres, 2002). Any other structures or functions responding to N and C availability, e.g. structural differences and capabilities to use organic N sources (Lilleskov *et al.*, 2011), are implicitly subsumed in the one of the two focal components to which they contribute the most and/or in the in the response of N uptake capacity to soil N availability (U_c, eq. S2).

Although fungal growth is assumed to be C limited it can also be simultaneously N limited indirectly, in the sense that N additions to the soil may (i) increase C export to EMF by plants and (ii) allow EMF to increase fitness by enhanced allocation to reproductive production at the expense of N uptake capacity.

Fungal competition for C and N

Whereas the above equations define physiology of EMF, to derive fungal strategy it is necessary to define functions for a single fungus (genotype, denoted by index i) in relation to its competitors (denoted by index o). Each fungus sharing a host plant with other fungi compete for C by individually increasing its N:C exchange rate with the plants (x_i, eq. 15) by adjusting its relative allocation to N uptake components (u_i) until its reproductive production (the fitness proxy, G_{fi}, eq. 14) peaks. G_{fi} is a function of the C use efficiency of growth (y_f) and three variable factors: the total plant C supply (C_f), the fraction of this C that is captured by the fungus (F_i, eq. 15) among its n_f competitors, and the fungus' allocation to reproductive production (1-u_i)

$$G_{fi} = y_f\, C_f\, F_i \left(1 - u_i\right) \quad \text{where} \tag{14}$$

$$F_i = \frac{x_i^z}{x_i^z + x_o^z (n_f - 1)} \quad \text{where} \quad x_i = \frac{N_{pi}}{C_{fi}} \quad \text{and} \quad N_{pi} = U_i - P_{fi}\, c_f \tag{15}$$

The partner discrimination parameter (z) determines how strongly the plants are able to prioritize fungi with higher x, which influences the N uptake benefits of EMF for the plants and thereby at what soil N availability plants switch between mycorrhizal and non-mycorrhizal strategies (Fig. S3). Although not addressed here, this trading strategy may have evolved by mechanisms of competition-based screening and partner fidelity feedback (Archetti *et al.*, 2011).

In addition to plant derived C, N uptake from soil (U_i) is also subject to inter-fungal competition (eq. 16), which we assume to depend on the number of fungal partners per tree (n_f) similarly to competition for plant derived C.

$$U_i = U[B_{fu}]\, n_f\, F_{ui} \quad \text{where} \quad F_{ui} = \frac{B_{fui}}{B_{fui} + B_{fuo}(n_f - 1)} \tag{16}$$

In eq. 16, U (eq. 1) is a function of the total biomass of EMF uptake components (B_{fu} , eq. 18), and F_{ui} is the fraction N uptake captured by fungus i, which depends on its individual uptake component biomass (B_{fui}). Using eq. 14, B_{fui} can be expressed as a function of G_f and u_i (eq. 17). B_{fu} (eq. 18) links fungal mean strategy (u) to plant C export (C_f).

$$B_{fui} = G_{fi} \frac{1 - u_i}{u_i} t_f \tag{17}$$

$$B_{fu}[u_i] = y_f\, C_f\, u \quad \text{where} \quad u = \frac{u_i}{u_i + \left(n_f + 1\right) u_o} \tag{18}$$

After sequentially inserting eqs. 17-18 in eq. 16, eq.16 in eq. 15, and eq. 15 in eq. 14, eq. 14 can be solved (numerically) for G_{fi}. The resulting G_{fi} (eq. 19) is a function of u_i and C_f, where C_f is determined by optimal plant behavior (eq. 22), thus linking fungal and plant behavior.

$$G_{fi} = G_{fi}[C_f, u_i] \tag{19}$$

Plant competition for N

While eq. 10 defines the fitness (net growth) of a plant in terms of its own properties, in order to define its fitness in the presence of competitors for soil N, we must account for an individual's uptake capacity (U_{ci}) in relation to the capacity of its competitors (U_{co}). Using eq.2, the uptake capacities are calculated from the plants' C export (C_f) and the resulting biomass of EMF uptake components (B_{fu}, eq. 18). The resulting N acquisition of an individual plant (N_{pi}, eq. 20) can be expressed as: The average uptake per area (N_p, eqs. 13, 21) times the number of competitors with overlapping uptake areas or common partner EMF (n_p), times the fraction N uptake captured by individual i (F_{pi}, eq. 21). Based on the clear-cut spatial separation and evidence of strong fungal discrimination among plant partners (Lekberg *et al.,* 2010) we assumed that competition is too efficient to allow any plant to pay less C per N received than other plants and thus will equalize N:C exchange rate (x) among plants. In effect, plants will compete via the amount of EMF supported by their C export and its uptake capacity. Thus, F_{pi} is equal to the uptake capacity contributed by individual (U_{ci}) relative to the total uptake capacity.

$$N_{pi} = N_p\, n_p\, F_{pi} \quad \text{where} \quad F_{pi} = \frac{U_{ci}[C_{fi},u]}{U_{ci}[C_{fi},u] + U_{co}[C_{fo},u](n_p - 1)} \tag{20}$$

$$N_p = N_p[U_c] \quad \text{where} \quad U_c = \frac{U_{ci}[C_{fi},u] + (n_p - 1)U_{co}[C_{fo},u]}{n_p} \tag{21}$$

Inserting N_{pi} (eq. 20) in eq.10, net growth of an individual plant among competitors (G_{pi}, eq. 22) can be expressed as a function of its C allocation to EMF (C_{fi}), and mean fungal allocation strategy (u) of the EMF.

$$G_{pi} = f[C_{fi}, C_{r0}, u] \tag{22}$$

In eq. 22, C allocation to non-mycorrhizal roots (C_{r0}) is zero except when evaluating a non-mycorrhizal strategy (see below).

Optimal (ESS) strategies of interacting plants and EMF

The behavior of plants and EMF are linked via their allocation strategies defined by C_{fi} and u_i, which determine fitness of plants (eq. 22) and EMF (eq. 19). The simultaneous optimization of plant and EMF behavior is done as follows: For each u_i the optimal C_f is calculated by maximizing eq. 22 with respect to C_{fi}, i.e. each plant individually increases its C export to the EMF (C_{fi}) to increase its N uptake (N_{pi}) until its net growth (G_{pi}) peaks, i.e. $\partial G_{pi}/\partial C_{fi} = 0$. In the calculation of G_p all individuals are identical ($C_{fi} = C_{fo}$) while the *change* in G_p, i.e. $\partial G_{pi}/\partial C_{fi}$, is evaluated with respect to only C_{fi} (and not C_{fo}). This resulting optimal C_f is a function of u_i and is inserted in the fungal fitness function (eq. 19). Optimal u is then calculated by maximizing fungal fitness (G_{fi}.) with respect to u_i. In the evaluation of G_{fi} all individuals are identical ($u_i = u_o$) while the *change* in G_{fi}, i.e. $\partial G_{fi}/\partial C_{fi}$, is evaluated with respect to only u_i (and not u_o). The optimal (ESS) values of C_f and u determines the system and all dependent variables.

Non-mycorrhizal plants

The physiological model for non-mycorrhizal plants differ from the mycorrhizal plant model only by the replacement of uptake via EMF with direct uptake by fine-roots ($C_f = 0$, $C_{r0} \neq 0$ in eq. 22) and the corresponding change in specific N uptake efficiency (e_u, Table S1). Competition for soil N among fine-roots of different plants is modeled in the same way as competition between different plants for the fungal derived N, but N_p, B_{fu} and C_f are replaced by U, B_{r0}, and C_r, respectively. We assumed that on average two plants compete for the same N (n_p= 2) in a non-mycorrhizal plant population.

Non-mycorrhizal versus mycorrhizal root strategies

A non-mycorrhizal strategy can invade a population of resident mycorrhizal plants if the increase in net growth per C invested is higher for a non-mycorrhizal root than for a mycorrhizal-root (including C allocation to EMF and supporting root structure), i.e. $\partial G_{pi}/\partial C_{ri} > \partial G_{pi}/\partial C_{fi} > 0$, where the last inequality follows from the definition of the ESS strategy of the resident mycorrhizal strategy. To calculate the N uptake due to the addition of a non-mycorrhizal root among existing mycorrhizal roots, the N uptake function (eq. 1) is extended by adding the uptake capacity of the non-mycorrhizal root to eqs. 20-21, in which U_c and F_{pi} are replaced by:

$$U_c = \frac{U_{ci}[C_{fi},u] + U_{ci}[C_{ri}] + (n_p - 1)U_{co}[C_{fo},u]}{n_p} \quad (23)$$

$$F_{pi} = \frac{U_{ci}[C_{fi},u] + U_{ci}[C_{ri}]}{U_{ci}[C_{fi},u] + U_{ci}[C_{ri}] + U_{co}[C_{fo},u](n_p - 1)} \quad (24)$$

The invasion potential $\partial G_{pi}/\partial C_{ri}$ is then evaluated as done in the calculation of optimal C_f of mycorrhizal plants based on eq. 22, where, after differentiation, C_{r0} is set to zero, which implies that the non-mycorrhizal root start growing at zero biomass.

The evaluation of the potential for a mycorrhizal strategy to invade a resident non-mycorrhizal strategy is analogous to the above, switching the positions of non-mycorrhizal and mycorrhizal N uptake in the equations.

Model parameterization

Physiological parameters typical of boreal pine forest were used (Table S1), with unknown parameters adjusted to match the range of measured productivities of EMF and wood in boreal forest soil N gradients (Fig. 2). Measured productivity of EMF represented only the upper 10 cm soil layer, while the total productivity is at least twice as high (Ekblad *et al.,* 2013), and was therefore multiplied by 2.5 to match our modeled effective soil depth of 25 cm. Modeled wood production was assumed to contribute 75% of total net growth. Because the specific N uptake efficiency (e_u) of EMF and fine-roots are unknown, a plausibly higher e_u for EMF than for plant roots was used (a factor of 300; Jennings, 1995; Smith & Read, 2008), which also enables the model to reproduce the commonly observed shift from mycorrhizal forest to non-mycorrhizal forest when going from low to high soil N availability (Taylor *et al.,* 2000; Högberg *et al.,* 2003; Kjøller *et al.,* 2012). Importantly, this parameterization does not build into the model any of the emergent results, such as for coexistence of strategies, symbiosis stabilization – soil N feedback, mycorrhizal colonization rates, effects of plant-fungal connectance, or effects of elevated CO_2. The (unknown) parameter *z* was set to *z*= 1 and tested for a range of higher and lower values (Fig. S3). The ranges of partner numbers used (connectance) are within observed ranges (Table S1). The effect of elevated atmospheric [CO_2] was modeled by increasing light saturated photosynthetic N use efficiency (*a)* by 30%, which lies within the range of observed effects in FACE experiments (Franklin, 2007).

Figure S2

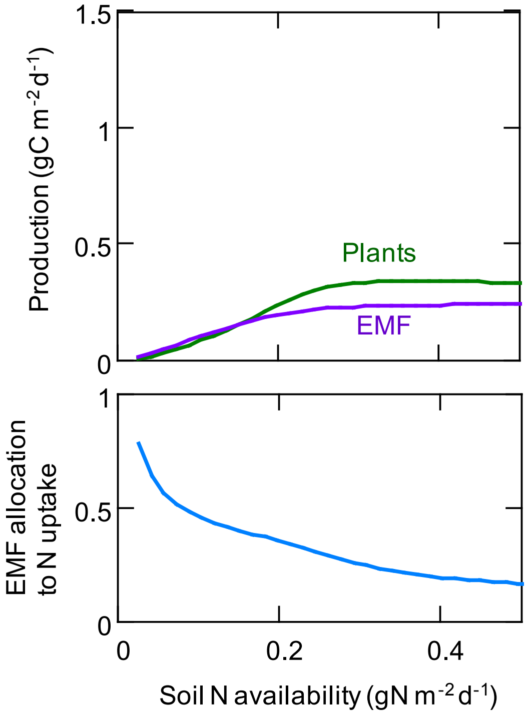

Figure S2. Modelled growth of plants (green lines) and ectomycorrhizal fungi (EMF, purple lines) and corresponding fungal strategy (fractional C allocation to N uptake (u), blue line lower panel) as functions of soil N availability, for connectance n_p = 8, and n_f = 1, i.e. no inter-fungal competition for C. In contrast to fig. 3, here the plants do not have the option to switch to a non-mycorrhizal strategy.

Figure S3

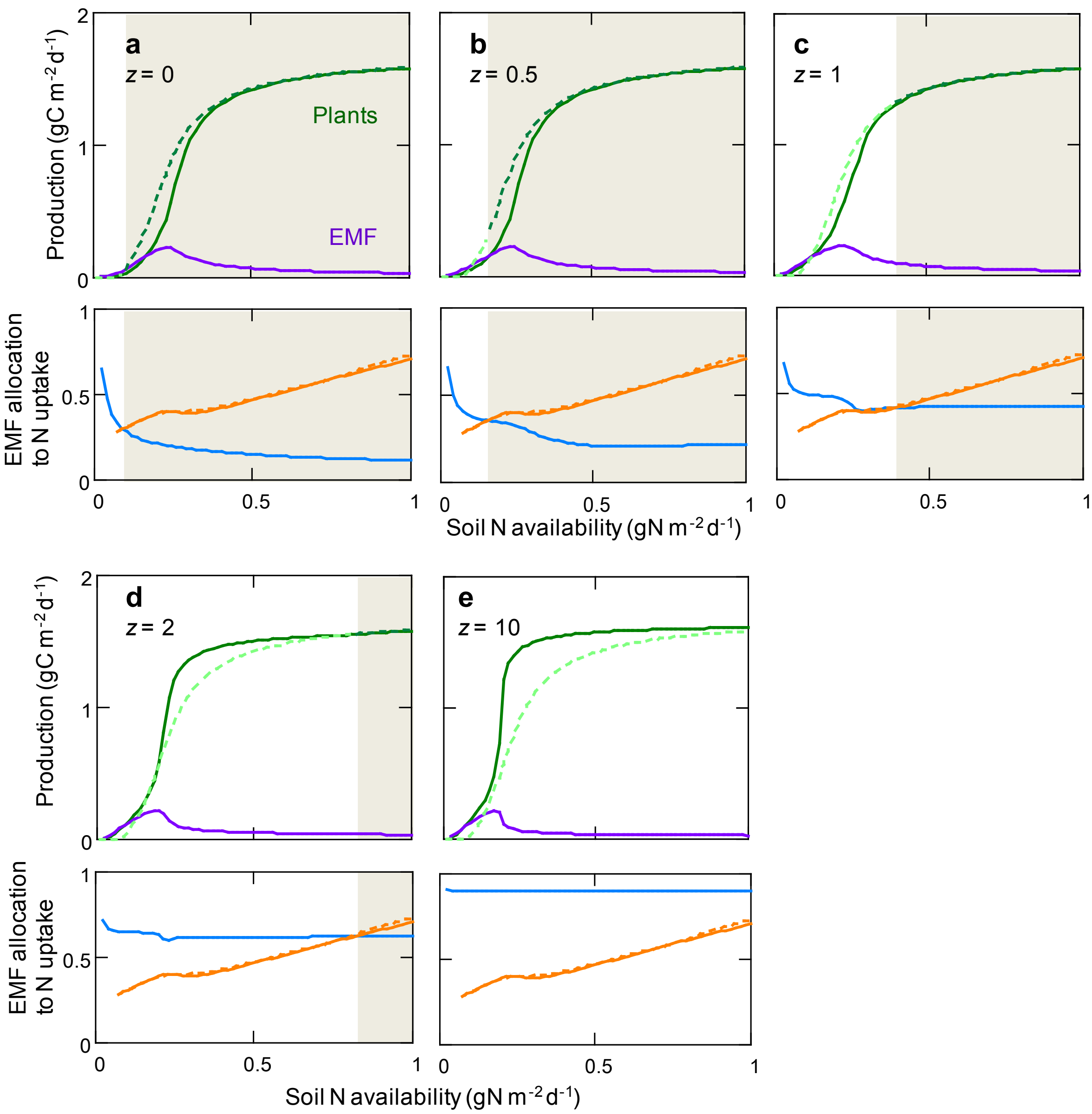

Figure S3. Modelled growth of plants (green lines) and ectomycorrhizal fungi (EMF, purple lines) and corresponding fungal strategy (fractional C allocation to N uptake (u), lower figures) as functions of soil N availability as shown in Fig. 2, but for different values of the partner discrimination parameter (z, Table E1, eq. 15). As soil N availability increases, fungal strategy (u) is first mainly determined by competition among EMF for plant C (blue line) and then (in shaded area) by competition with a non-mycorrhizal root strategy (solid orange line). The intensity of inter-fungal competition for plant C increases with z, resulting in higher fungal allocation to N uptake (u) and higher plant N uptake, which delays the shift from a purely mycorrhizal to a mixed mycorrhizal and non-mycorrhizal plant strategy (shaded area) as soil N availability increases. Fungal –plant connectance were n_f= n_p= 8 in all panels.

References (that are not included in the main text)

Archetti M, Scheuring I, Hoffman M, Frederickson ME, Pierce NE, Yu DW. 2011. Economic game theory for mutualism and cooperation. *Ecology Letters* **14**(12): 1300-1312.

Choudhury BJ. 2001. Implementing a nitrogen-based model for autotrophic respiration using satellite and field observations. *Tropical Ecology* **2**: 141-174.

Lilleskov EA, Hobbie EA, Horton TR. 2011. Conservation of ectomycorrhizal fungi: Exploring the linkages between functional and taxonomic responses to anthropogenic N deposition. *Fungal Ecology* **4**(2): 174-183.

Sinsabaugh RL, Manzoni S, Moorhead DL, Richter A. 2013. Carbon use efficiency of microbial communities: stoichiometry, methodology and modelling. *Ecology Letters* **16**(7): 930-939.

Wong SC, Cowan IR, Farquhar DG. 1979. Stomatal conductance correlates with photosynthetic capacity. *Nature* **282**: 424-426.
